# A toolkit of thread-based microfluidics, sensors, and electronics for 3D tissue embedding for medical diagnostics

**DOI:** 10.1038/micronano.2016.39

**Published:** 2016-07-18

**Authors:** Pooria Mostafalu, Mohsen Akbari, Kyle A. Alberti, Qiaobing Xu, Ali Khademhosseini, Sameer R. Sonkusale

**Affiliations:** 1 Nano Lab, Department of Electrical and Computer Engineering, Tufts University, Medford, MA 02155, USA; 2 Biomaterials Innovation Research Center, Division of Biomedical Engineering, Department of Medicine, Brigham and Women’s Hospital, Harvard Medical School, Cambridge, MA 02139, USA; 3 Harvard-MIT Division of Health Sciences and Technology, Massachusetts Institute of Technology, Cambridge, MA 02139, USA; 4 Wyss Institute for Biologically Inspired Engineering, Harvard University, Boston, MA 02115, USA; 5 Laboratory for Innovation in MicroEngineering (LiME), Department of Mechanical Engineering, University of Victoria, Victoria, BC V8P 2C5, USA; 6 Department of Biomedical Engineering, Tufts University, Medford, MA 02155, USA; 7 Department of Physics, King Abdulaziz University, Jeddah 21589, Saudi Arabia

**Keywords:** thread diagnostics, 3D microfluidics, implantable devices, wearable devices, flexible sensors, flexible electronics, pH sensor, strain sensor, glucose sensor

## Abstract

Threads, traditionally used in the apparel industry, have recently emerged as a promising material for the creation of tissue constructs and biomedical implants for organ replacement and repair. The wicking property and flexibility of threads also make them promising candidates for the creation of three-dimensional (3D) microfluidic circuits. In this paper, we report on thread-based microfluidic networks that interface intimately with biological tissues in three dimensions. We have also developed a suite of physical and chemical sensors integrated with microfluidic networks to monitor physiochemical tissue properties, all made from thread, for direct integration with tissues toward the realization of a thread-based diagnostic device (TDD) platform. The physical and chemical sensors are fabricated from nanomaterial-infused conductive threads and are connected to electronic circuitry using thread-based flexible interconnects for readout, signal conditioning, and wireless transmission. To demonstrate the suite of integrated sensors, we utilized TDD platforms to measure strain, as well as gastric and subcutaneous pH *in vitro* and *in vivo*.

## Introduction

Implantable diagnostic devices (IDDs) and smart wearable systems (SWSs) that are capable of *in situ* sample collection and integration with the complex three-dimensional (3D) structure of biological tissues offer significant opportunities for diagnosing and treating diseases. Recent technological advances in the miniaturization of sensors and the fabrication of smart materials have progressively changed the landscape of health care by providing the ability to develop devices that can continuously monitor a patient’s health status^1–5^. Examples of such devices are electrocardiogram electrodes, temperature sensors, pH sensors, and flexible batteries that have been used for real-time health monitoring^6–12^. The development of such devices requires overcoming the challenges associated with the mismatch between the mechanical and topographical properties of the semiconductor-based electronics and the biological tissues. Flexibility and biocompatibility are other key characteristics needed for devices in these applications.

Materials such as polyimide^13^ and parylene^14^ have been extensively used as substrates for IDDs and SWSs. However, device microfabrication on such materials is expensive because it requires clean room facilities and specialized processing. Paper has emerged as a promising substrate for implantable devices and wearable electronics because of its universal availability, low cost, environmental friendliness, and ease of fabrication^15–17^. Several paper-based platforms have been developed recently, such as lateral flow immunoassays for the detection of analytes^18,19^, detection of DNA or proteins^20^, and electrochemical biosensing^21,22^. Recently, nanofibrous polymeric substrates have been developed to fabricate elastic and flexible electronics that can be sutured^3^. The fibrous microstructure of the substrate enables gas and liquid permeation and promotes the ability to pattern metallic electrodes on them.

Although these substrates hold great promise for the creation of wearable and implantable devices, their overall structure and form has essentially remained two-dimensional, limiting their function to tissue surfaces, such as skin. However, the ability to integrate functional components, such as sensors, actuators, and electronics, in a way that they can penetrate multiple layers of tissues in a 3D topology would be a significant advance. For example, wound fractures and orthopedic implants, which have complex 3D structures, would greatly benefit from the implantation of physical (for example, strain) and chemical (for example, pH) sensors that can monitor the local tissue environment and provide valuable information to optimize patient-specific treatments. IDDs and SWSs can also benefit from a system that enables the delivery of bodily fluids from different parts of the same tissue or from different tissues to the sensing elements. Such a system for acquiring complex-sensing information distributed in space and time can provide more useful information about the function of an individual organ. Microfluidic systems are promising platforms that allow the manipulation of minute amounts of liquid (~nl) in small footprints^23^. The ability to fashion microfluidic networks for spatio-temporal chemical analysis in a tissue or organ environment in 3D would represent a significant advance. Microfluidic systems with integrated sensors have been widely used to perform molecular assays on blood samples^24^, capture circulating tumor cells^25^, and detect cancer-specific biomarkers^26^. However, these systems are mainly limited to planar structures and are able to transport liquid over small distances only.

The overarching goal of this paper is to develop a toolkit of miniaturized sensors, electronics and microfluidic networks that addresses these challenges for the next generation of IDDs and SWSs, using thread as a substrate for a completely thread-based diagnostic device (TDD) platform. Threads are naturally thin and flexible and can be easily manipulated into complex shapes using well-known textile processing methods. Threads can be derived from natural materials, such as cotton and silk, or can be made using synthetic biomaterials on a large scale using well-known spinning processes^27^. The mechanical and degradation properties of threads can be modified by changing the material composition^28^. Such properties make threads an excellent choice for the development of IDDs and SWSs. Threads also have the potential for complex 3D physiochemical analysis due to the ability to suture them in three dimensions through multiple layers of tissue. They can be used as microfluidic channels for injection and delivery of analytes by exploiting the natural capillary action, which is similar to paper-based microfluidic devices^29,30^. The ability of a thread to be formed into an arbitrary 3D structure provides natural 3D microfluidic channels that, along with thread-based sensors and electronics, can provide a 3D analytical platform.

From the fabrication standpoint, photolithography, screen-printing, and stamping methods are common approaches for the implementation of electrodes on planar substrates, which have also been used to realize functional sensing ‘tattoos’ on the body^2,5,31^. In these methods, the size of the electrodes depends on the feature size on the photo mask, stencil or shadow mask. When working with threads, the fundamental limit is set by the thread diameter, which has already reached sub-micron dimensions using conventional threading processes. The micropatterning of such threads using current textile technology, such as sewing, knitting, and embroidering to make functional devices justifies the scalability of thread-based devices for different sizes and shapes for various applications.

**Figure 1:**
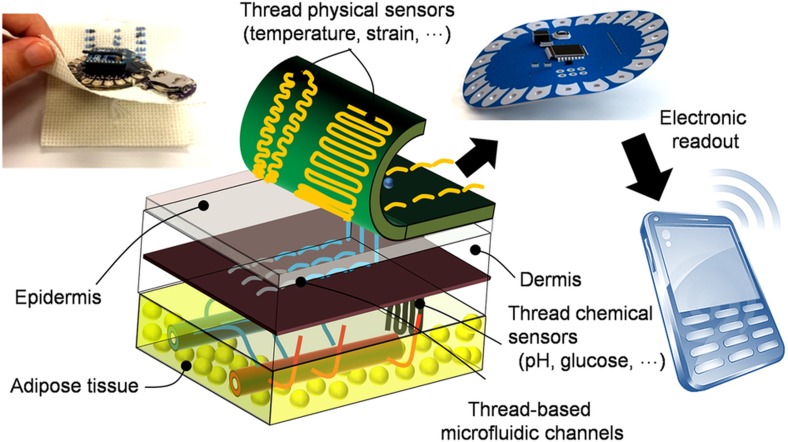
A toolkit of thread-based chemical and physical sensors, microfluidic channels, and interconnects for the realization of a thread-based diagnostic device (TDD), shown here for transdermal health monitoring.

Our TDD is based on the use of functional threads as building blocks, which are inherently flexible and can be fashioned on any flexible substrate or sutured into any biological tissues in an arbitrary 3D geometric form. We propose the fabrication of threads with different physical, chemical, and biological functions to serve as sensors, microfluidics, and electronics, and to be integrated as a TDD (Figure 1). Hydrophilic threads were embroidered onto a highly hydrophobic woven fabric to serve as microfluidic channels for the controlled delivery of bodily fluids to the sensing zones. Conductive threads infused with nanomaterials, such as carbon nanotubes (CNTs), carbon nanopowders, polyaniline (PANI) and their combination, were used as thread-based electrodes for the *in vitro* and *in vivo* measurement of glucose, pH, temperature, and strain as representative physiological properties. Outputs of the sensors were connected to readout electronics on a different layer, which consisted of electronics for signal processing and wireless communication to a smartphone or a computer using conductive threads as interconnects.

Although prior studies regarding thread as a substrate focused exclusively on one aspect, such as microfluidics or sensors^29,32^ with a single thread type for *ex vivo* low-cost diagnostic applications, we demonstrate an integrated TDD platform for implantable applications with a diverse and greatly expanded toolkit of sensors, electronics, and microfluidic functions, such as custom-designed nano-infused threads as multiple chemical and physical sensors.

## Materials and methods

Phosphate-buffered saline solution (PBS), hydrochloric acid (HCl), and sodium hydroxide (NaOH) were purchased from Sigma Aldrich, MA, USA. For preparation of the electrodes, carbon ink (E3456 Graphite Erconinc, MA, USA), silver/silver chloride ink (AGCL-675C, Conductive Compound, Hudson, NH, USA), functionalized CNTs (Sigma Aldrich), and PANI emeraldine base (Sigma Aldrich) were obtained. Polydimethylsiloxane (PDMS; Sigma Aldrich) and blue insulator (E6165, Erconinc, MA, USA) were utilized as dielectric covers of the electrodes.

### Doping of the PANI

The PANI was doped based on a previously published procedure^33^. A PANI base (500 mg) was added to 20 ml of HCl (0.1 M). The solution was mixed for 5 h at −4 °C inside an ice bath. The PANI-covered thread was kept in a desiccator before usage.

### CNT ink

CNTs functionalized with a carboxylic group were purchased from Sigma Aldrich. The CNTs were first premixed in IPA (2 mg ml^−1^) and stirred by a vortex, and then they were homogenized in an ultrasonic bath.

### Scanning electron microscopy

Scanning electron microscopy (SEM) images were acquired using FESEM ultra55 (12 kV). The microparticles were dried on a paper substrate, mounted on aluminum stubs using conductive carbon paint and sputtered for SEM analysis.

### Characterization of sensors

Characterization of the strain sensor was performed using a micro Instron 5542 mechanical tester (Norwood, MA, USA) with a 1-kN load cell. The two ends of the thread with a length of 15 mm were connected to moving stages, and uniform strain was applied, whereas the resistance was measured using a source meter (Keithley source meter 2400). Data were acquired after the output data were stabilized.

The pH- and temperature-sensing measurements were conducted using a customized potentiometric board using off-the-shelf components, including an LMP91200 as a main front-end amplifier component. For the pH readout, the voltage generated after transmission through an ultralow noise buffer was sampled and digitized by a microcontroller and then transmitted to an XBee LilyPad or Bluetooth low-energy (BLE) wireless module, and the data were collected in real-time on a smartphone or computer. The BLE package for cell phone communication is shown in Supplementary Figure S9.

Glucose sensing measurements were also performed utilizing a customized printed circuit board with off-the-shelf components, including a TI LMP91000 as a main component of the low-power potentiostat. The LMP91000 applied a voltage of 0.5 V to the glucose sensor, taking the current from the glucose sensor as input and generating analog voltage as an output. The output voltage, similar to the pH readout after sampling and digitization using the microcontroller, was sent to a wireless transmitter.

### *In vivo* measurement and animal protocol

The research protocol was approved and in compliance with Tufts University’s Institutional Animal Care and Use Committee (IACUC, protocol #M2013-53) in accordance with the Office of Laboratory Animal Welfare at the National Institutes of Health. Briefly, animals were anesthetized using 2–3% isoflurane inhalation, and the incision site was shaved and cleaned with alternating povidone–iodine and ethanol scrubs. For subcutaneous pH measurements, a small incision (<1 cm) was made, and the probes were inserted through the wound. Alternatively, two 18-G needles were inserted under the skin, and the probes were fed through them, ensuring that the ends of the two probes did not touch each other. For gastric pH measurement, the probes were fed through two 16-G oral gavage needles (Braintree Scientific, Braintree, MA, USA) and inserted through the mouth into the stomach. Last, for pH measurements of the blood, the animals were sacrificed via thoracotomy while under anesthesia, and blood samples were taken from the heart and placed into green-top blood collection tubes containing lithium heparin (Becton Dickinson).

## Results and discussion

### Fabrication of functional threads

Physical and chemical sensors, a microfluidic network, and interconnects are three components of the envisioned 3D TDD platform for intimate tissue integration. Functional threads, such conductive threads, nano-infused threads and hydrophilic threads, serve as building blocks for the realization of these components.

**Figure 2:**
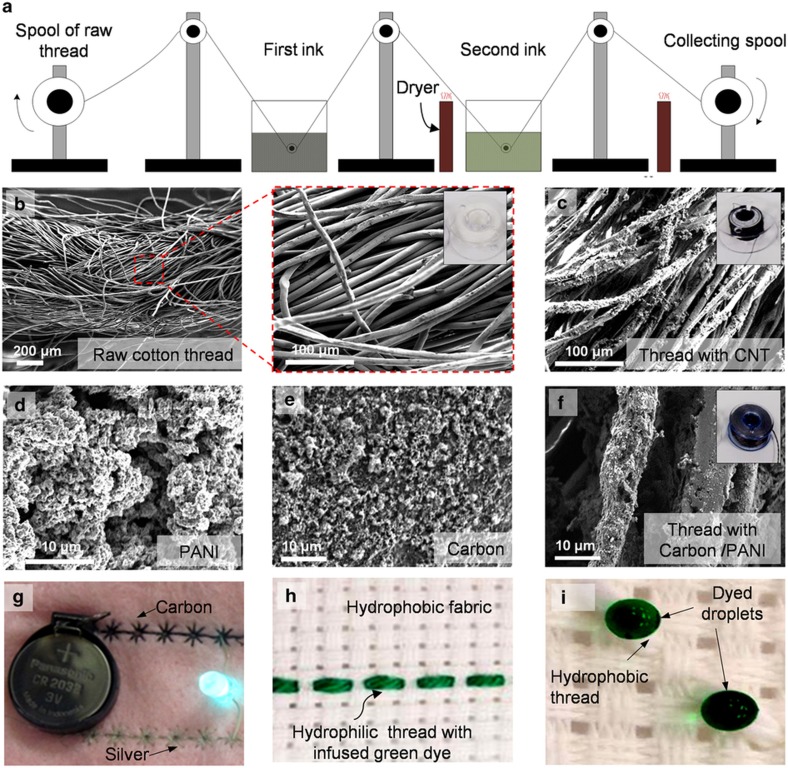
Fabrication of functional threads. (**a**) Schematic representation of the coating system for preparation of conductive threads. (**b**) SEM image of the cotton raw thread. (**c**) SEM image of a nano-infused thread coated with CNTs. (**d**–**f**) SEM images of the surface of PANI-, carbon-, and carbon/PANI-coated threads. (**g**) Pattern conductive threads as interconnects on a woolen fabric to illuminate an LED. (**h**) Embroidered hydrophilic threads on a hydrophobic fabric after green dye was infused. (**i**) Hydrophobic threads repelling water. Green food dye was used to improve visualization. LED, light-emitting diode.

Electrodes, which are the key constituents of any chemical and physical sensors, were fabricated by sequentially passing the core cotton threads through multiple wells containing conductive inks (Figure 2a). Such a device was previously developed by our group to coat cell-laden hydrogels on suturing threads^34^. Conductive inks explored in this work include silver/silver chloride, carbon, CNTs, and PANI prepared as described in the experimental section (Figures 2b–h). A dryer was utilized to cure the coating layer on the thread whenever needed. In addition, an ultraviolet light was used for sterilization in animal studies. An image of the setup is shown in Supplementary Figure S1, and colorful dyes are added for better visualization. Meters of functionalized threads were fabricated using the proposed manufacturing method and collected on rotating spools (Figures 2b, c, and f).

For strain sensors, nano-infused threads were made by coating CNT and PDMS layers on elastic threads (polyurethane (PU) threads). The use of PDMS enhanced the mechanical integrity of the conductive layer and reduced the occurrence of delamination. For pH sensors, the working electrode was made from nano-infused threads coated with carbon and PANI, and the reference electrode was made from silver/silver chloride (Supplementary Figure S2). PANI was chosen because of its biocompatibility, high electrical conductivity, and superior stability in electrolytes^35^. Because it has acid/base-dependent oxidation states, it is an ideal material for pH sensors. Moreover, PANI forms a thin layer with a 3D network of interconnected nanofibrils (Figure 2d) that promotes its mechanical flexibility and enhances the mechanical integrity of the coated layer. Carbon is selected because it enhances the electrochemical reactivity and promotes electron transfer due to its unique physical and chemical structures^36^. Silver/silver chloride is a very common reference electrode for stabilizing the voltage level of the analyte solution. More conductive and dielectric threads are shown in Supplementary Figure S2.

The biocompatibility of the carbon/PANI, silver/silver chloride, and nafion utilized for the fabrication of chemical sensors has already been demonstrated in prior work^37^. Physical sensors were covered with layers of PDMS, which is a well-known biocompatible material. Cotton and polyurethane threads have also been shown to be biocompatible in previous studies^38^. Moreover, we also performed additional experiments to test the biocompatibility of cotton thread by culturing 3T3 cells (Supplementary Figure S3). This demonstrated that cotton thread has excellent cell viability even after 7 days.

To demonstrate the compatibility of the fabricated conductive threads with current textile technologies, a programmable embroidering machine (Brother PE-500) was used to pattern conductive threads on a woolen fabric to illuminate a light-emitting diode with a coin cell battery (Figure 2g).

### Wicking properties of threads

Cotton threads commonly used in the apparel industry are often coated with wax and other additives to facilitate gliding and to prevent the thread from tearing during the textile process^39^. Despite the thin wax layer on their surface, we noticed that the as-received threads have some wicking properties. This may be because the wax layer only covers a thin layer on the surface of the thread and does not fill all the gaps between the strands. However, such fibers are not ideal for efficient wicking of liquid using capillary action. We used plasma cleaning (50 W for 2 min) to remove the wax layer from the fiber strands, rendering them more hydrophilic. Oxygen plasma removes the wax from the thread surface and adds the –OH bonds on the surface of the thread. As an alternative, if we intended to eliminate capillary action and render the threads hydrophobic, then we dipped the as-received threads into a commercially available silicone lubricant. The water repellant material covered the surface of the thread and filled the pores between the strands, blocking any possible liquid flow through them. We embroidered hydrophilic threads on a hydrophobic woven fabric (Figure 2h) to form patterned microfluidic channels for liquid delivery. We confirmed that the liquid flow was restricted to within the patterned threads as channels and did not wick onto the water-repellent fabric (Supplementary Figure S4f).

**Figure 3:**
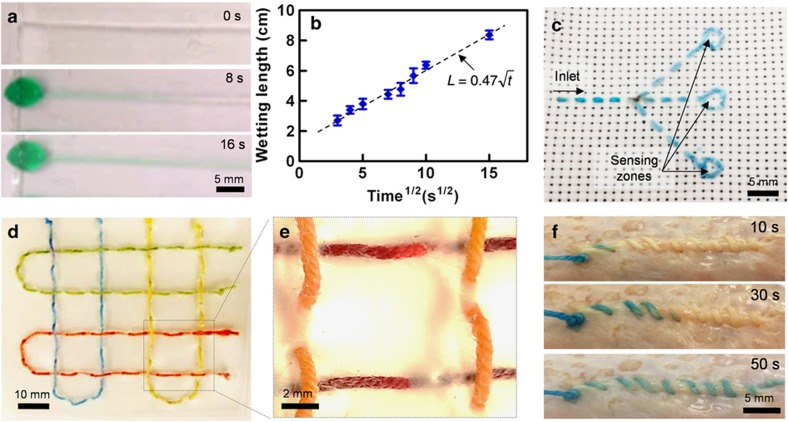
Threads as microfluidic channels or flow carriers. (**a**) Image sequences extracted from a video illustrating the flow of a green dye in a plasma-treated cotton thread due to capillary action. (**b**) Wetted length as a function of the square root of time. (**c**) A microfluidic flow splitter embroidered on a hydrophobic fabric for chemical sensing. (**d**) A 3D microfluidic network pattern created by sewing a thread onto a PET film. Three colored fluids were wicked into the system without mixing. (**e**) Close-up views of overlapping threads showing no mixing of two different fluids. (**f**) Wicking of a blue dye into a thread patterned on a chicken skin. PET, polyethylene terephthalate.

The wicking property of the plasma-treated cotton threads was characterized by measuring the speed of the water-filling front (Figures 3a and b). The flow followed the standard Washburn relationship in which the filling length is a function of the square root of time^29,30,40,41^ under controlled environmental humidity (relative humidity ~50%). Evaporation has a significant effect on the wicking properties of the threads^41^. Evaporation can also affect the concentration of the analytes reaching the sensing zone and may cause errors in the sensing results. However, for implantable devices, where the microfluidic system is embedded within tissue (which has a less differential saturated vapor pressure and comprises a more water-rich environment) evaporation has less of an impact.

We created a passive three-way microfluidic splitter by embroidering plasma-treated threads on a hydrophobic woven fabric (Figure 3c). Such a microfluidic splitter enables the delivery of samples to three different sensors. In addition, we showed the ability to fabricate a topologically complex 3D microfluidic system for transporting liquid in three dimensions by sewing a hydrophilic thread in a polyethylene terephthalate (PET) film (Figures 3d and e). Such a system can be used to deliver different samples on a single platform. To demonstrate the ability of the thread-based microfluidic system to deliver samples in biologically relevant substrates, a hydrophilic thread was sutured on a chicken skin (Figure 3f). The sample wicked along the suture without significant leakage due to the presence of a fat layer on the chicken skin. Capillary forces in the thread exist even after the initial thread wetting, which is also promoted by the diffusion effects. See Supplementary Figure S5 for the fluidic flow after thread saturation by a liquid. Colorful dyes were used for better visualization. Supplementary Figure S5a shows an optical image of the thread after the addition of two subsequent dyes, and Supplementary Figure S5b shows that the flow rate is reduced by almost half after the thread gets saturated, but the thread is still viable as a microfluidic channel. According to the Washburn equation, the fluidic flow rate in a known time is a function of the capillary diameter, viscosity of the liquid, and contact angle of the liquid with the thread. Moreover, diffusion is another factor governing the flow of species and is a function of the concentration gradient across the thread. The capillary diameter is expected to decrease due to the particle accumulation from the longer duration flow, consequently reducing the flow rate over time. However, in our case, because we are only interested in facilitating the transfer of small sensing species, such as glucose and hydrogen ions, we believe that such a flow will be sustained over a longer duration for different biological fluids (blood, serum, urine, and so on).

### Physical sensors

Physical parameters such as pressure, stress, strain, and temperature are important indicators of tissue health. Here we developed strain and temperature sensors as representative thread-based physical sensors for implantable devices.

**Figure 4:**
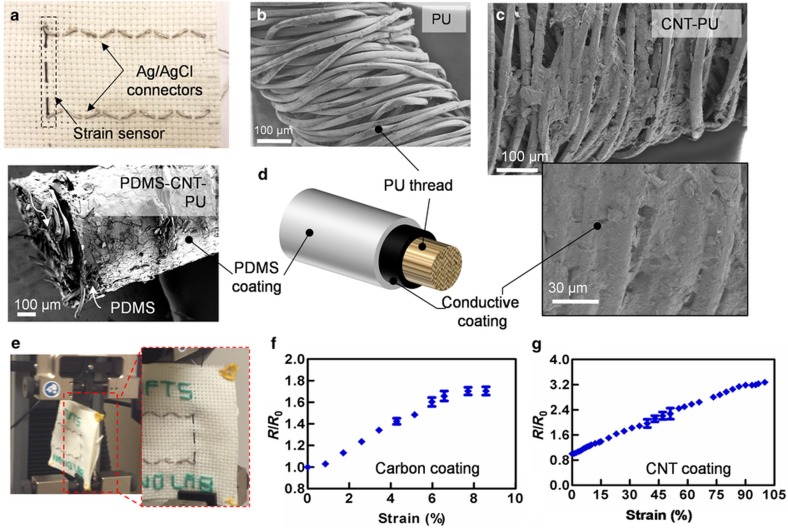
Thread-based strain sensor. (**a**) Embroidered strain sensor and interconnected wirings on a woven fabric. SEM images of (**b**) an uncoated stretchable thread, (**c**) a thread coated with CNT (the inset shows a higher magnification of the highlighted area), and (**d**) the sandwich structure of the PU-CNT-PDMS. (**e**) Optical images showing the setup used for stretching the sample (the inset shows a close view of the setup under testing conditions). (**f** and **g**) Variation of the relative resistance as a function of the strain for threads coated with (**f**) carbon ink and (**g**) CNT, respectively.

The basic operation of strain sensors is based on a variation of electrical parameters, such as resistance because of mechanical deformation. The gauge factor (GF), that is, the relative change in electrical resistance due to a mechanical strain, the response time, and the maximum detectable strain are critical parameters for the evaluation of strain sensors. A typical GF and maximum strain for conventional strain sensors are ~2 and ~5%, respectively^42^. Strain sensors were fabricated from PU threads coated with carbon ink and CNTs. Such materials are biocompatible and have been extensively used for the fabrication of strain sensors^12,43,44^; they have been suggested as promising candidates due to their high elasticity and sensitivity^12,45^. PU is a thermoplastic that can be chemically activated when plasma-treated. We observed that conductive threads made from PU have superior conductivity compared with other types of elastic threads. Carbon ink and CNTs, which were functionalized with carboxylic groups, bind easily to plasma-treated threads using the method described previously. A thin layer of PDMS was coated on the threads to protect the conductive layer from scratching and delaminating during cyclic stretching loads^45^. In addition, the integrity of the system in the sandwich structure and the strong adhesion between CNTs and the two elastic layers prevented buckling and fracture of CNTs, providing excellent linearity and elasticity. The stretchable conductive threads were then embroidered on a woven construct and connected to an electronic readout circuitry using silver/silver chloride conductive threads (Figure 4a). Figures 4b and c show SEM images of the uncoated and conductive threads, respectively. The conductive particles covered most of the surface of the thread and infiltrated its pores, creating interconnected conductive materials. Although some particles are attached to the outer layer, most of them are attached to the core thread, preventing them from breaking under higher strain. Figure 4d illustrates the sandwich structure of the CNT between the PU thread and PDMS, confirming the complete coverage of the surface. Moreover, PDMS, as a dielectric material, has an important role in isolating the conductive thread from other wiring in the system.

To evaluate the performance of the strain sensors, they were stretched at a constant rate (0.1 mm min^−1^) with a tensile machine (Figure 4e). Strain gauges registered linear changes in electrical resistance when the sensor was stretched. Figures 4f and g show the variation in the relative resistance versus strain for threads coated with carbon ink and CNTs, respectively. The results indicate that the strain sensors made from CNTs were capable of measuring higher strains (up to 100%, GF~3) compared with those made from carbon ink (strains up to 8%, GF~2). This is because CNTs possess higher deformability due to their fibrous structure^12^. CNTs also offer faster response times and lower creep^12^.

Another important marker for monitoring tissue health is temperature, as temperature variations can be indicators of inflammation or bacterial infection^46^. Moreover, temperature measurement is crucial for precise sensing in integrated systems as most chemical and physical biosensors are temperature dependent. Nickel and platinum are the best choices for materials among the readily available metals for resistive temperature sensors. Moreover, they have a linear temperature coefficient of resistance over a long temperature range. However, coating the threads with such materials using inexpensive methods is not feasible. Here we used CNT-coated threads as resistive temperature sensors. Forty centimeters of thread were embroidered in a meandering zigzag pattern on a woven fabric. Such a pattern provided an electrical resistance of 100 Ω in a small footprint. Supplementary Figure S6 shows a linear change in resistance for temperatures ranging from 20 to 40 °C, which is in the biological range of most viable tissues.

### Chemical sensors

pH is one of the most important parameters in the body and is an established indicator of health. Almost all biochemical processes in the body are affected by pH. For example, the pH of a wound is an indication of the wound condition and can be correlated to angiogenesis, protease activity, and bacterial infection^47,48^. The healing process occurs more readily in an acidic environment; however, extremely acidic pH values can indicate a bacterial infection^47^. Thus, monitoring the pH of the tissue may provide a method for measuring the condition of the wound bed and ultimately enables determination of the wound’s response to treatment. Another application for pH sensing is in the gastric environment, which is essential for the diagnosis of gastrointestinal diseases, such as inflammatory bowel disease and gastroesophageal reflux disease, or infection from *Helicobacter pylori*^49–51^.

**Figure 5:**
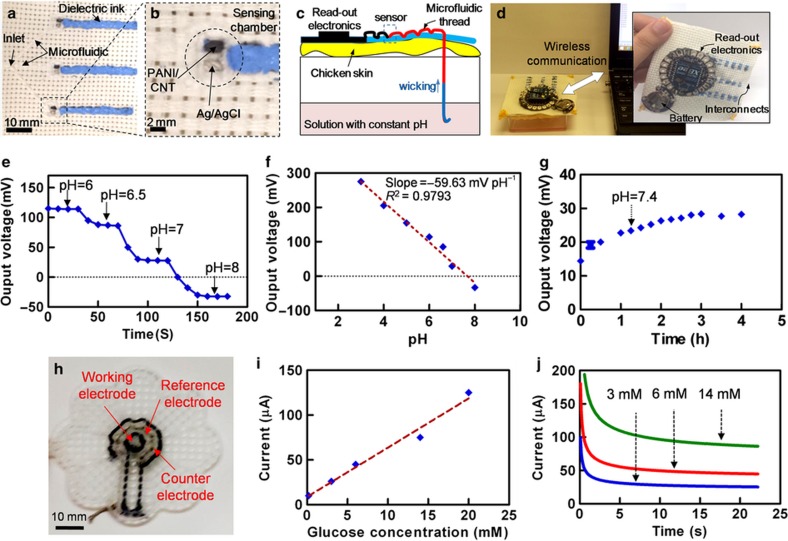
Characterization of chemical sensors. (**a** and **b**) Optical image of a multiplexed microfluidic pH sensors assay. (**c**) Schematic illustration of measuring pH in an *in vitro* skin model. (**d**) Sensing system communicating with an external computer via a wireless system. (**e**) Transient response of the pH sensor to different pH values. (**f**) Calibration plot of the pH sensor. (**g**) Continuous pH measurement for four hours. (**h**) Optical image of the glucose sensor. (**i**) Calibration plot of the glucose sensor. (**j**) Transient response of the glucose sensor to different glucose concentrations in the PBS solution. PBS, phosphate-buffered saline.

Here we present a thread-based pH sensor consisting of conductive threads and a microfluidic splitter with three channels for the delivery of a sample to the sensing chambers (Figures 5a and b). The microfluidic splitter was fabricated by patterning hydrophilic threads on a hydrophobic woven fabric. For pH measurements, a potentiometric approach was used. In this method, the open-circuit potential of a working electrode was measured with respect to a reference electrode. CNT coated with doped PANI and silver/silver chloride threads served as the working and reference electrodes, respectively. To evaluate the silver/silver-chloride and doped PANI as electrodes, their conductivity was measured. To perform the experiment, the cotton threads were covered by silver/silver-chloride and doped PANI via several repetitions of dipping and drying. Then, the impedance was measured between two ends. The result is shown in Supplementary Figures S8a, b. Conductivity and charge accumulation on the working electrode are dependent on the protonation and deprotonation of the doped PANI under different pH conditions. The output voltage correlates to the pH value according to the Nernst equation:

$$E=E_{0}-KT/e\left(pH\right)$$

where *E*_0_ is the standard reduction potential of hydrogen with respect to the reference electrode silver/silver chloride, *e* is the electron charge, and *K* and *T* are the Boltzmann constant and the temperature, respectively.

The pH was measured in various buffer solutions with pH values ranging from 3 to 8. All the solutions were prepared using hydrochloric acid and sodium hydroxide in PBS^52^. This range covers the physiological pH that occurs in the body. Before measurement, each sensor was immersed in purified water to ensure similar starting conditions. The pH of the solution was independently measured using a pH meter (Benchtop pH/MV Meter—860031). A hydrophilic thread was used to deliver the liquid sample from a buffer solution reservoir to the microfluidic thread channel. The thread was passed through a chicken skin to mimic the subcutaneous measurements; the results demonstrate the confinement of biological fluids in the thread-based microfluidic channel without significant leakage onto the chicken fat (Figure 5c and Supplementary Figure S7). Recorded data were sent wirelessly to a desktop computer (Figure 5d). Figure 5e shows the stability of the pH measurement at different acidic/basic conditions. A rapid response time (<30 s) was achieved as the signal was stabilized in a few seconds after changing the pH. The voltage was found to be linearly dependent on the pH value with the slope of −59.63 mV per pH, which exhibits near-ideal Nernstian behavior (Figure 5f). To assess the long-term signal stability, the voltage drop was measured in a solution with a pH of 7.4 for 4 h. A 2.5-mV h^−1^ drift was observed, indicating the high stability of the fabricated pH sensors (Figure 5g).

The concentration of glucose is an important indicator of diabetes and has to be tightly monitored for the management of this disease. The ability to monitor the glucose level *in vivo* over a long time would enable the intensive control of blood glucose concentration in patients with diabetes. In addition, such long-lasting implantable sensors reduce the frequency of implantation and replacement, resulting in less discomfort for the patients. Measuring glucose using thread-based sensors is promising for long-term monitoring as threads can be implanted in the body with minimum invasiveness.

Here we developed an amperometric glucose sensor consisting of carbon/functionalized CNT threads (working electrode), carbon threads (counter electrode), and silver/silver chloride threads (reference electrode). Threads were patterned onto a woven fabric as shown in Figure 5h. Glucose oxidase enzyme solution (20 μl, 1 mg ml^−1^) and 2 ml of nafion (5%) were subsequently added to the working thread. Nafion was used for immobilization of the enzyme^53^. Glucose solutions with concentrations in the range of 2–15 mM were prepared in PBS with a pH of 7.4. Potassium ferricyanide in KCl (100 mM) was added to the solution as a mediator. Chronoamperometry was used for glucose measurements. Briefly, two pulses with voltages of 0.5 and 0 V with a 50% duty cycle were applied, and the output current was measured. Figure 5i shows the sensor response to different concentrations of the glucose in the solution. Extremely rapid responses (~ms) were observed, with the amplitude depending linearly on the glucose concentration (Figure 5j).

### *In vivo* evaluation of the integrated thread-based microfluidics, sensors, and electronics

#### Integrated system

A sensing patch using the thread-based toolkit was developed. It consisted of a thread-based microfluidic system with integrated physical and chemical sensors, as described earlier, and a separate readout electronics module. The integration of different components was easily achieved through sewing to form a TDD. The readout electronic circuitry consisted of data acquisition from the sensors, followed by amplification and wireless transmission of the data to a cell phone or computer. A set of off-the-shelf components, including the Texas Instruments LMP91200 and LMP91000 (Texas Instrument, Dallas, TX, USA), was used for measurement of the pH and strain. For measurement of pH-sensing results, the output voltages of the electrodes were attached to an ultralow input current bias buffer amplifier. The output analog voltage was read by an Arduino (SparkFun, Niwot, CO, USA) microcontroller with a 1-Hz sampling frequency. The data were sent to an XBee (SparkFun, Niwot, CO, USA) wireless transmitter. The electronics were implemented by sewing the circuit board on the fabric. Although the board itself was rigid in this initial prototype, it was small and externally placed to not influence the sensor performance. Moreover, one can easily port the electronics onto a flexible printed circuit board on a polyimide substrate through commercial vendors or explore emerging paper/textile substrates^54–56^.

#### *In vivo* measurements

**Figure 6:**
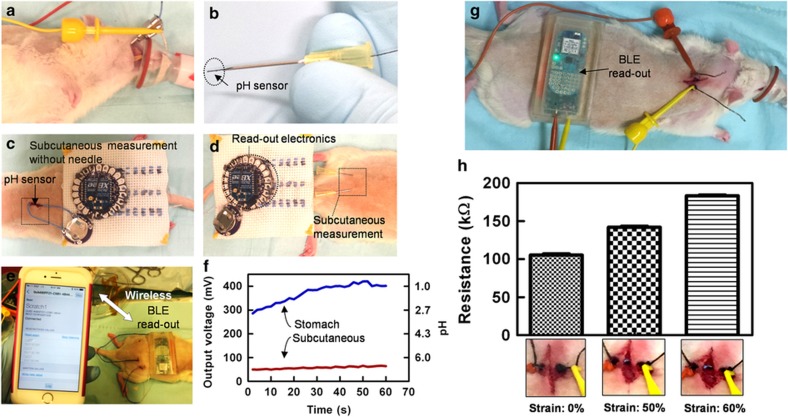
*In vivo* measurement of pH, both subcutaneously and in the stomach. (**a**) The view of a thread-based sensor inserted into the stomach of a rat via the mouth using an oral gavage needle as a guide. (**b**) pH sensor passed through a needle before subcutaneous implantation. (**c** and **d**) Implanted sensors connected to the patch. (**e**) pH-sensing system communicating with a smart phone via a Bluetooth platform. (**f**) pH readings of the sensors in the stomach and under the skin. (**g**) *In vivo* measurement of the strain under three different conditions: relaxed; medium stretch; and high stretch. (**h**) Measured strain signal under various wound conditions.

The gastric and subcutaneous pH values were measured as a representative form of chemical sensing *in vivo*. For gastric pH measurements, the thread-based sensor was directly inserted into the stomachs of animals (rats) using oral gavage needles as guides (Figure 6a). Subcutaneous measurements were performed by implanting the electrodes under the skin (Figure 6c), or by placing them under the skin using needles (Figure 6d). The results obtained from measuring the pH subcutaneously and in the stomach are shown in Figure 6f. The pH measured under the skin was neutral and stable over 1 min of measurement (pH=7.0±0.1). As expected, the pH value in the stomach was highly acidic and fluctuated from 1 to 3 over time.

The *in vivo* measurement of the pH was recorded for a short period of time. It demonstrated the feasibility of the thread-based pH sensors as implantable devices. The characterization of the pH sensor in Figure 5g shows the stability and the drift of the sensor over time. The performance of the sensor might degrade when used continuously for an *in vivo* application due to the adsorption of proteins and other molecules. Antifouling-coating material will be necessary to improve the lifetime of the sensor, but this is not the goal of this study, and an excellent review on the options available can be found elsewhere^57^.

Suture strain measurements were performed on three wound conditions to mimic the wound closure process during healing: (i) closed; (ii) semi-closed; and (iii) open. For this measurement, a 1-cm incision was made on the back of the neck. The strain sensor was then passed through the wound and secured with a simple knot on each side. Strain-monitoring equipment was then attached using alligator clips. For different wound conditions, the wound site was closed manually, and the strain was measured. The recorded data were then transferred to a mobile phone wirelessly and saved for further analysis. Figure 6h shows the typical values measured by this system for the wound closure model. Note that the strain of the wound was created manually; therefore, the indicated values in the *x* axes are exact numbers, matching the value taken from the calibration plot of the strain sensor.

## Conclusion

We have developed a toolkit of thread-based devices to measure physical (strain and temperature) and chemical (pH and glucose) markers in an integrated TDD platform. *In vivo* functionality of the system was evaluated by measurement of pH and strain in different parts of the body. The primary benefit is that threads are low in cost and are biocompatible, having been widely used in the apparel industry. The electrical and surface properties of the threads were tailored to transport fluids using capillary action and were infused with nanomaterials to perform electrochemical sensing using an inexpensive dipping approach. The performances of sensors were evaluated and optimized individually. The integrated system was used to measure pH and strain *in vitro* and *in vivo*. Our research suggests that TDD has the potential to act as a part of human skin or clothing and to even be implanted. The ability to suture TDD intimately into a tissue or organ in three dimensions adds a unique feature that is not available with other flexible diagnostic platforms. We believe that such a TDD could eventually find a wide range of applications, such as smart sutures for surgical implants, smart bandages to monitor wound healing, integration with textile or fabric as personalized health monitors and point-of-care diagnostics, and embedding into engineered tissue constructs for organ-on-a-chip platforms. We envision being able to extend the approach to more than the strain, pH, or glucose sensors mentioned here by functionalizing them with sensing chemistries to measure proteins, DNA and other biomarkers directly in the tissues where they are implanted. Although we demonstrated thread-based interconnects, future efforts could be in the area of integrating other electronic components, such as capacitors, diodes, and transistors, on threads, which will result in a truly self-contained integrated platform with unmatched size, flexibility, and maneuverability. For thread-based devices to be used as long-term implantable devices, additional studies on the biocompatibility of the proposed cotton-based threads and their functionalized forms may be required. Such studies may indicate that threads elicit an immune response when used long term, which would necessitate exploration of other biocompatible materials for thread creation that have a more favorable immune response.
