# Supplementary material for: A toolkit of thread-based microfluidics, sensors, and electronics for 3D tissue embedding for medical diagnostics
Source: Microsyst Nanoeng. 2016 Jul 18;2:16039. doi: 10.1038/micronano.2016.39 (PMC6444711; doi:10.1038/micronano.2016.39)
Supplement: Supplementary Information [file micronano201639-s1.pdf]

Supplementary file

A toolkit of thread-based microfluidics, sensors, and electronics for 3D tissue embedding for medical diagnostics

Pooria Mostafalu[1], Mohsen Akbari[2,3,4,5], Kyle A. Alberti[6], Qiaobing Xu[6], Ali Khademhosseini[2,3,4,7] and Sameer R. Sonkusale[1]

Microsystems & Nanoengineering (2016) **2,** 16039; doi:10.1038/micronano.2016.39; Published online: 18 July 2016

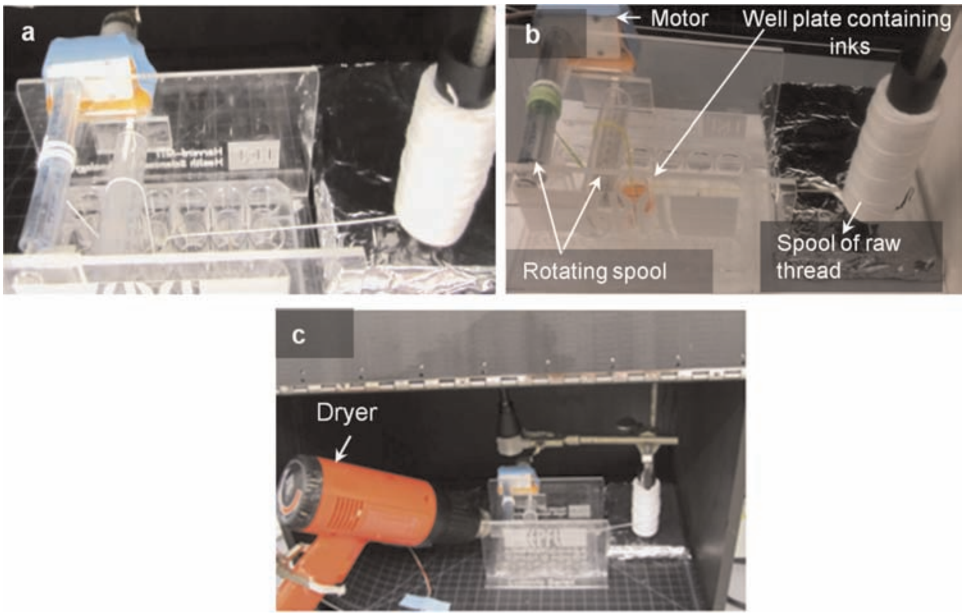

Figure S1 (**a**) Images of the setup for coating the threads. (**b**) Two different colorful dyes were added for better visualization. (**c**) Image of setup in box with a dryer.

[1]Nano Lab, Department of Electrical and Computer Engineering, Tufts University, Medford, MA 02155, USA; [2]Biomaterials Innovation Research Center, Division of Biomedical Engineering, Department of Medicine, Brigham and Women's Hospital, Harvard Medical School, Cambridge, MA 02139, USA; [3]Harvard-MIT Division of Health Sciences and Technology, Massachusetts Institute of Technology, Cambridge, MA 02139, USA; [4]Wyss Institute for Biologically Inspired Engineering, Harvard University, Boston, MA 02115, USA; [5]Laboratory for Innovation in MicroEngineering (LiME), Department of Mechanical Engineering, University of Victoria, Victoria, BC V8P 2C5, USA; [6]Department of Biomedical Engineering, Tufts University, Medford, MA 02155, USA and [7]Department of Physics, King Abdulaziz University, Jeddah 21589, Saudi Arabia.
Correspondence: Sameer Sonkusale (sameer@ece.tufts.edu)

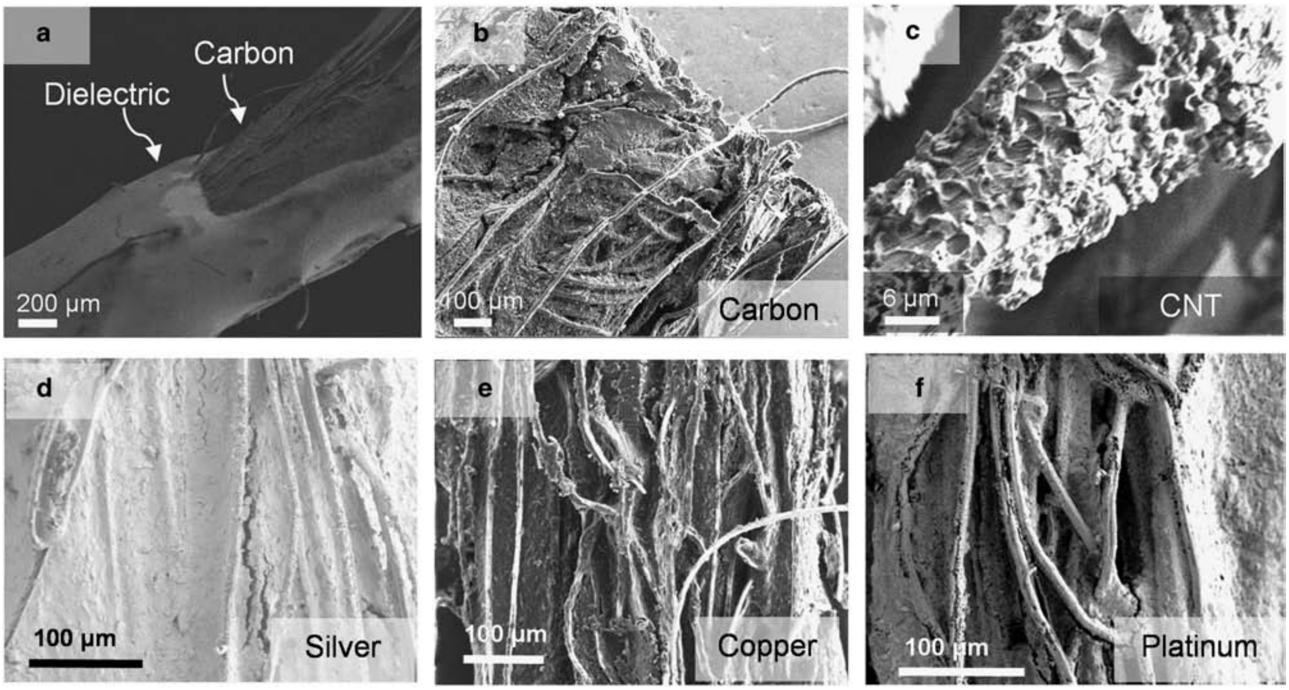

Figure S2 (**a**) SEM image of thread coated with dielectric and carbon. SEM image of thread coated with (**b**) carbon. (**c**) CNT. (**d**) Silver. (**e**) SEM image of copper electroplated on carbon thread. (**f**) SEM image of platinum electroplated on carbon thread.

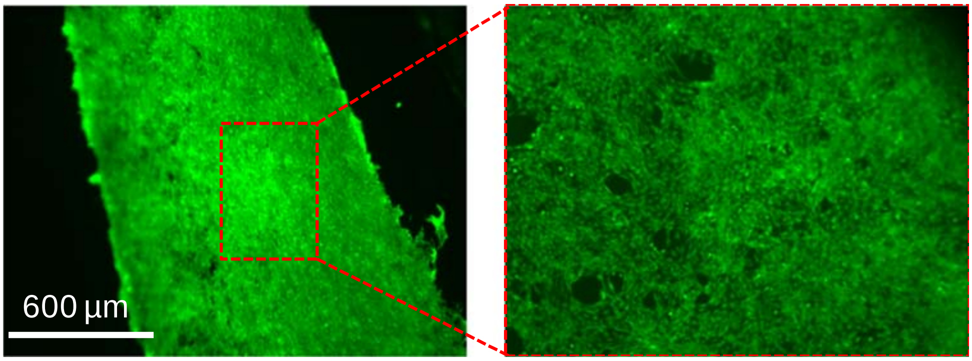

Figure S3 Live/Dead® cell viability of the 3T3 cells after 7 days showing live cells stained with Calcein-AM (green).

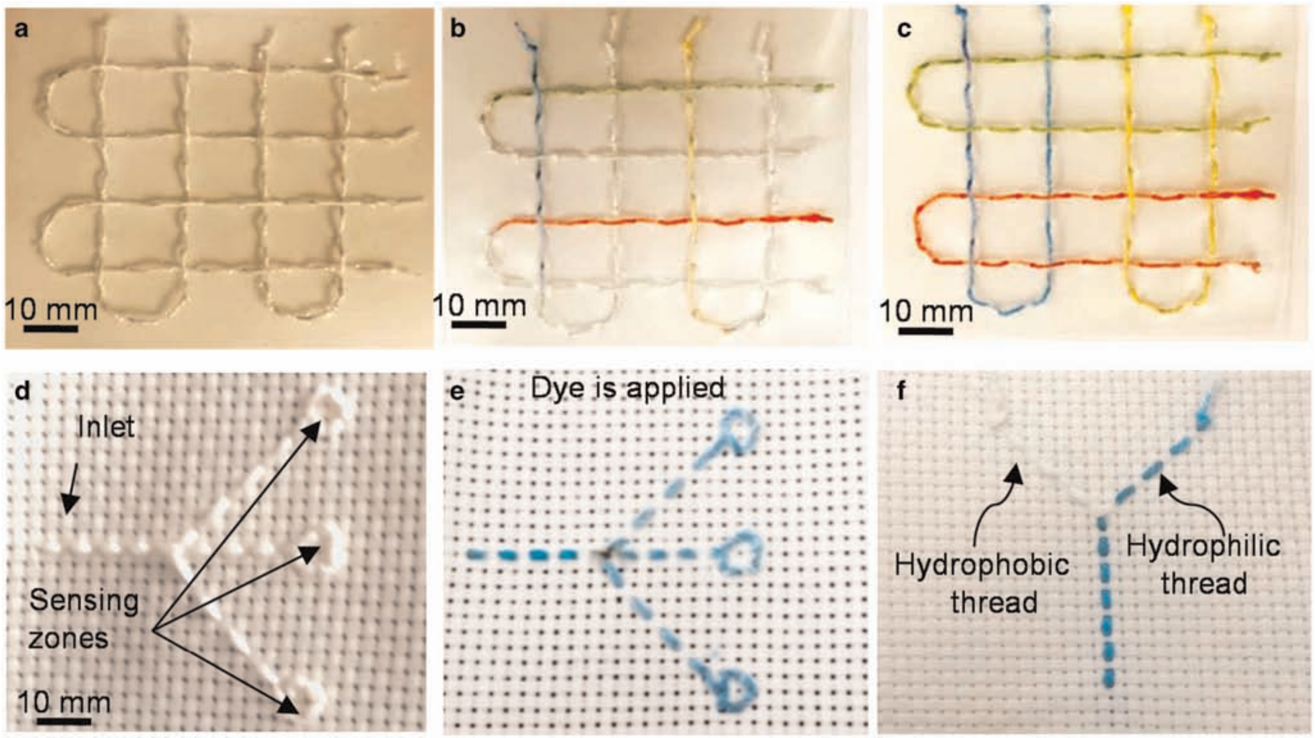

Figure S4 Threads as microfluidic channel for liquid delivery. (**a**) Sewed thread on transparent sheet (PET) without dye. (**b**) Optical image of the network when different dyes were applied. (**c**) Optical image of the network when dye was completely wicked. Optical image of the multiplexed sewed cotton threads on a woven fabric (**d**) without dye and (**e**) when dye is applied. (**f**) Optical image of a thread-based microfluidic system with hydrophilic and hydrophobic branches. A solution of blue food dye was added to the hydrophilic thread for clarification.

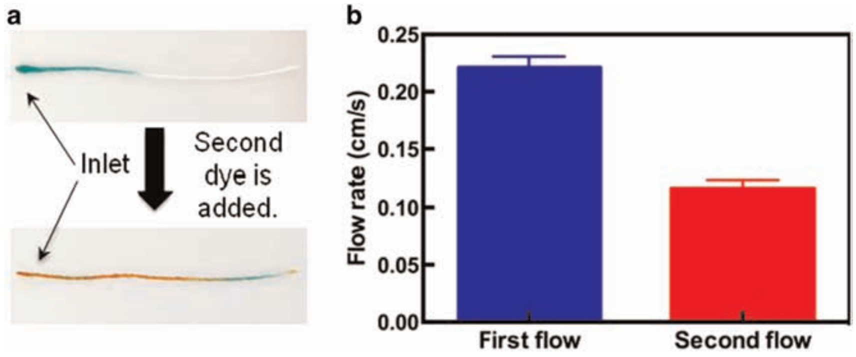

Figure S5 Fluidic flow after thread saturation. (**a**) optical images after addition of subsequent analyte. (**b**) Flow rate comparison after initial wetting of a cotton thread.

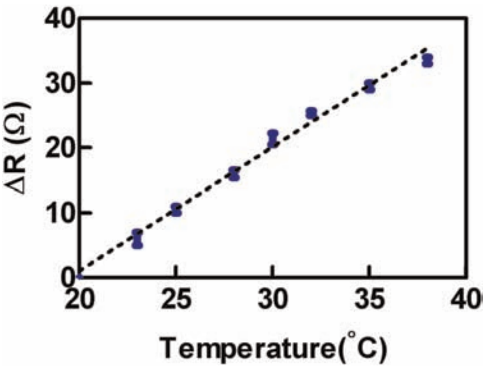

Figure S6 Calibration plot of the temperature sensor.

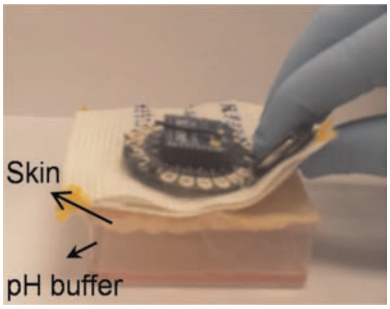

Figure S7 Experimental setup for characterization of the pH sensor.

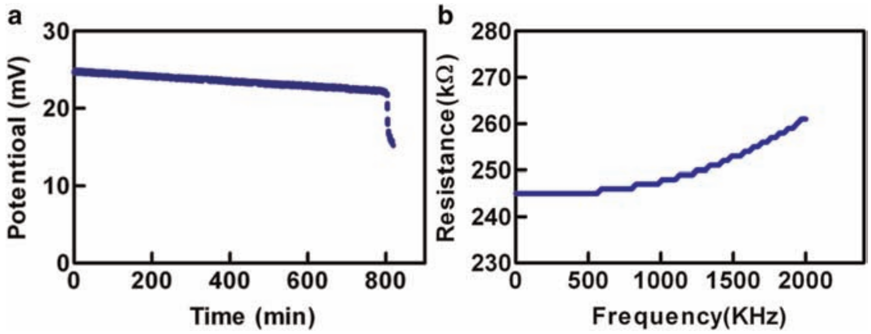

Figure S8 (**a**) Characterization of reference electrode with respect to commercial reference Ag/AgCl electrode. (**b**) Doped PANI characterization.

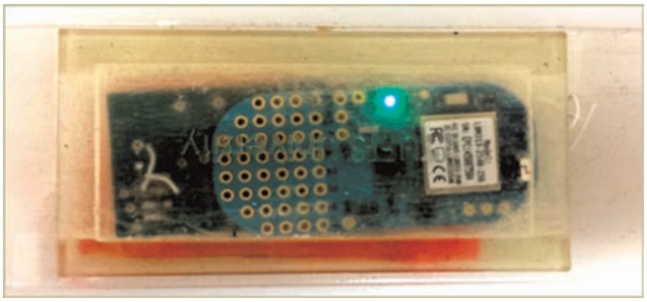

Figure S9 Wireless dressing using BLE module for the measurement of the pH and temperature.
